# Bone cells modulate *Cutibacterium acnes* colonization and biofilm formation on bone and titanium

**DOI:** 10.1016/j.bioflm.2026.100404

**Published:** 2026-10-05

**Authors:** Jennifer Varin-Simon, Christine Guillaume, Yasser Dghoughi, Renaud Siboni, Frédéric Velard, Sophie C. Gangloff, Marius Colin, Fany Reffuveille

**Affiliations:** a Université de Reims Champagne-Ardenne, BIOS, Reims, France; b Université de Reims Champagne-Ardenne, UFR Pharmacie, Reims, France; c Université de Reims Champagne-Ardenne, UFR Odontologie, Reims, France; d Université de Reims Champagne-Ardenne, UFR de Médecine, Reims, France; e Centre Hospitalier Université de Reims, Service de Chirurgie Orthopédique et Traumatologique, Reims, France

**Keywords:** *Cutibacterium acnes*, Bone explant, Biofilm, Titanium, Prosthetic joint infection

## Abstract

*Cutibacterium acnes* causes around 10 % of prosthetic joint infections, predominantly in the shoulder. Its eradication is complex due to its low virulence and its ability to form biofilm. Although *C. acnes* behavior on prosthetic materials like titanium is described, its interactions with bone remain poorly understood. This study investigated the impact of bone on *C. acnes* adhesion and biofilm formation.

Two clinical strains, C2 (non-PJI) and PJI2 (PJI-isolate), were incubated with fresh (cellularized) or frozen (cell-free) bone explants, fibronectin-coated titanium discs, and combinations thereof. Adhesion and biofilm formation were assessed by colony counts, microscopies, and RT-qPCR of biofilm-related genes.

Results showed that *C. acnes* adhered and initiated biofilm formation to bone, with upregulation of stress- and biofilm-related genes (*groEL*, *tuf*, *fbaA*, *eno*, *sucD*) in C2. Osteoblasts promoted *C. acnes* adhesion to bone, as evidenced by the reduced bacterial attachment observed on cell-free bone. The presence of bone affected *C. acnes* behavior on titanium: osteoblast and their secretions enhanced bacterial adhesion to titanium with an effect depending on the bacterial strain as well as the osteoblast donor. The bone mineral matrix also influenced bacterial behavior, increasing the adhesion of the PJI2 strain to the implant. However, the composition of the biofilm matrix on titanium remained unchanged.

In conclusion, the bone microenvironment promotes *C. acnes* adhesion and biofilm formation on both bone and titanium, especially through bone cells presence. This effect strongly depends on a strain-osteoblast donor combination, highlighting the necessity of new diagnostic approach for *C. acnes* prosthetic joint infections.

## Highlights

• ***C. acnes* adheres to bone**.
• **Bone impacts on *C. acnes* implant colonization**.
• **Individual variability** highlights specific risks of biofilm formation.

## Graphical abstract

**Figure:**
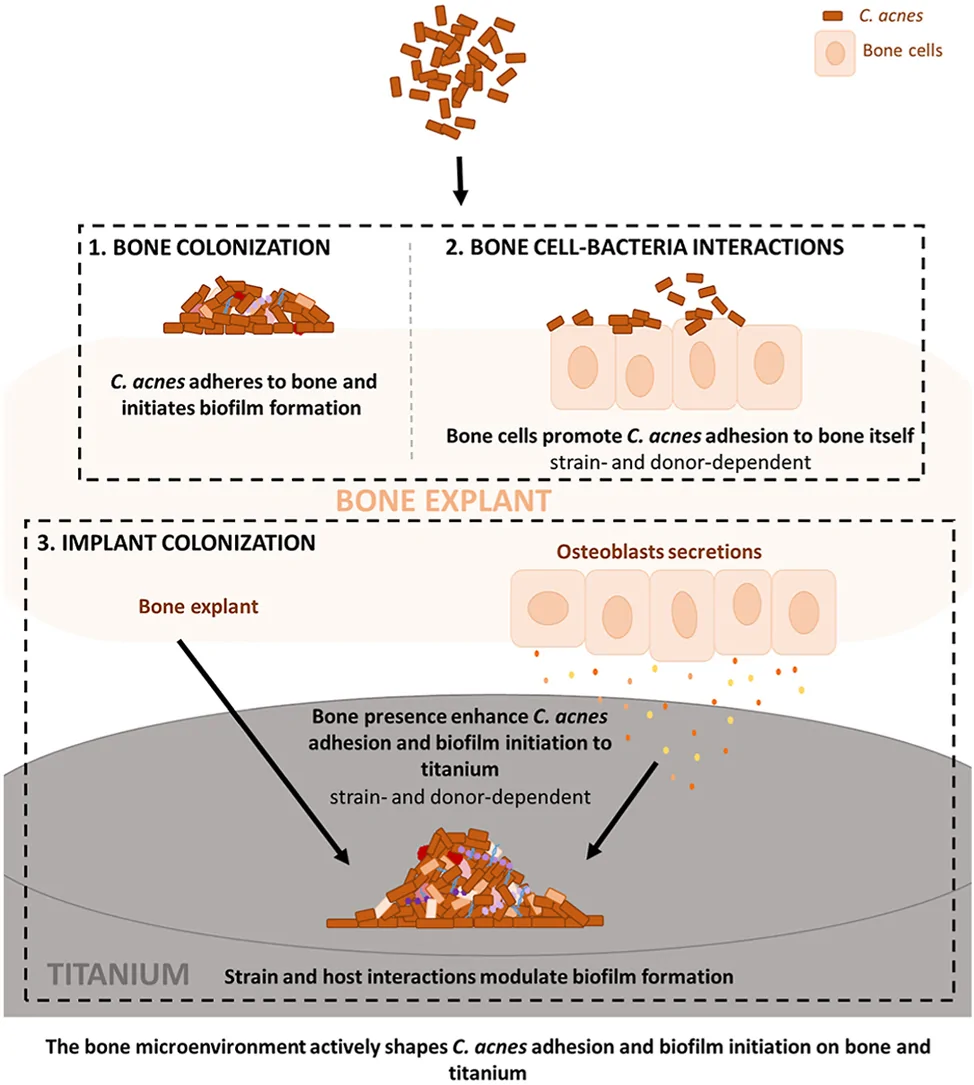

## 1 Introduction

Despite preventive measures, 1-2 % of primary arthroplasties lead to bacterial infections [1]. *Cutibacterium acnes*, a Gram-positive skin bacterium, is involved in approximatively 10 % of prosthetic joint infections (PJIs) with a marked tropism for shoulder sites [2,3]. The weak or absent inflammatory response complicates the diagnosis of *C. acnes* infections. It is likely that *C. acnes* develops bacterial persistence and initiation of biofilm formation in this context [4].

Biofilm is a complex community of bacteria exhibiting different metabolic states, glued in a matrix composed of exopolysaccharides, proteins and extracellular DNA, conferring a favorable microenvironment in which bacteria can escape host immune system and antibiotic treatment [[5], [6], [7]]. Different studies showed that *C. acnes* can adhere and form biofilm on surfaces often used in prosthesis manufacturing: titanium alloys (TiA), cobalt-chromium-molybdenum (CoCrMo), polyether ether ketone (PEEK) and stainless steel [8,9]. In a recent study, our team highlighted differences in *C. acnes* biofilm formation on titanium alloy compared with plastic, emphasizing the need to develop a model representative of the physiological context of prosthetic joint infections (PJIs) to better understand the mechanisms of *C. acnes* biofilm formation [10]. *C. acnes* is also able to adhere to biotic surfaces, especially through binding to proteins (e.g. fibronectin) present in bone tissue, which rapidly cover the material in *vivo* [11,12].

While *C. acnes* adhesion to protheses had been investigated, its behavior toward bone has not yet been thoroughly investigated. This appears as crucial investigations to conduct as *C. acnes* presence within bone tissue has been observed, notably in osteocytes lacunar-canalicular networks (OLCN), in an implant-associated osteomyelitis murine model [13].

Bone is composed of mineral and organic phases. The mineral part represents 70 % of the bone and is mainly composed of hydroxyapatite, while the organic part is mainly composed of type I collagen fiber, non-collagen proteins, proteoglycans and glycoproteins, and cells [14]. Bacteria can interact with mineral and/or organic bone molecules to promote colonization and induce prosthetic joint infections. Indeed, studies have shown that bacteria, such as *S. aureus*, can interact with hydroxyapatite, collagen, fibronectin, sialoproteins but also with bone cells like osteoblasts [15,16]. Regarding the latter, bacteria such as *S. aureus* can adhere to osteoblasts by interaction between fibronectin-binding protein (FnBP) present on *S. aureus* cell wall, fibronectin, and α5β1 integrin (fibronectin-binding osteoblast receptor), leading to bacterial internalization [17]. Further studies demonstrated that *C. acnes* is also capable of entering the intracellular compartment of osteoblasts [18]; Mongaret *at al*., 2020] as well as bone marrow-derived mesenchymal stem cells [19], although the underlying mechanism remains unknown. These findings indicate that *C. acnes* can interact with bone cells.

While the presence of bone explant has already been shown to modulate *S. aureus* adhesion and biofilm formation on titanium [20], no studies have been carried out on the influence of bone and its components on the pathophysiology of *C. acnes* infection. The aim of this study was to observe and characterize the impact of bone tissue and its various compounds on *C. acnes* adhesion and biofilm formation abilities.

## 2 Materials and methods

### 2.1 Bacterial strains and culture

Two clinical *C. acnes* strains named C2 and PJI2 were obtained from the bacteriology virology parasitology and mycology department of Reims University Hospital Center (CHU Reims). C2 is a non-PJI-related strain, isolated in the context of a surgical procedure but not associated with any infection and in consequence, considered to represent skin contamination, and PJI2 was isolated from shoulder prosthetic infection. The origin and MLST profile of these two strains have been previously described [10].

*C. acnes* was cultured on Columbia agar supplemented with 5 % of sheep blood (BioRad, Hercules, CA, USA) under anaerobic atmosphere using the GENbag system (Biomerieux, Marcy l’Etoile, France), at 37°C, for 5 days. For all assays, one colony of *C. acnes* strain was deposited in 1.5 mL-tube previously filled with Brain Heart Infusion (BHI) broth (BioRad, Hercules, CA, USA). After a 5-days incubation at 37°C, absorbance at 600 nm was measured and adjusted with medium to 1 before dilution at 1:100 in diluted BHI (dBHI: 90 % sterile water and 10 % BHI) and 1 mL was inoculated in 24 well-plates with different supports (bone explants, titanium coated with fibronectin, and combination of both). The dBHI medium mimics a nutrient-depleted environment, as encountered in the bone–prosthetic environment. This medium promotes biofilm formation despite a reduction in bacterial growth and adhesion to the titanium disc [10]. For osteoblast secretion assays, 500 μL of supernatant was first added to each well, followed by 500 μL of bacterial suspension to obtain a final 1:100 bacterial dilution in the well. Plates were incubated at 37°C, for 5 days, under anaerobic conditions.

### 2.2 Titanium coating

In this study, titanium alloy (TiAl6V4) discs with a diameter of 1.18 cm, in accordance with the medical standard ISO 5832-3 (or ASTM F136), were shot-peened by CRITT-MI (Charleville-Mézières, France). Briefly, disc surface was treated by microbead projection and roughness was controlled. The shot-peened titanium disc obtained was then coated with bovine fibronectin (Sigma-Aldrich, St. Louis, MO, USA) and named “titanium fibronectin” (TF) discs. For that, a drop of 200 μL of 100 μg/mL fibronectin solution was deposited on the titanium disc surface and incubated 2 h at room temperature. After washes in PBS 1X, titanium discs were dried overnight.

### 2.3 Bone explants and osteoblasts cultures

Bone explants were obtained from Orthopedic and Trauma department of Reims University Hospital, from patients having undergone arthroplasty and given their written informed consent (authorization and registration number DC-2014-2262 provided by the French Ministry of Research).

Bone explants were processed in the laboratory immediately after arthroplasty. Bone fragments were obtained using a 2-mm diameter trocar, or a 11-mm diameter hole saw for recellularization assays.

A portion of the bone fragments was used immediately as fresh (FreB) or after 3 freezing cycles as frozen bone (FroB). Frozen explants were prepared by subjecting samples to 3 freeze (−80°C)/thaw (room temperature) cycles of at least 4 h each, to eliminate viable human cells. According to the equation established after bone microcomputed tomography by Lamret et al., each bone explant was weighed to calculate the total accessible area through weight-area equivalence: Bone area (cm^2^) = [[Weight (mg) – 6.7323]/0.1866] x 100 [20].

The remaining explants were used for the isolation of primary human osteoblasts. Primary human osteoblasts were obtained by migration from bone explants placed in culture as previously described [21]. Briefly, bone explants were washed in PBS 1X before being digested in a solution of 0.5 % trypsin-EDTA (Gibco, Paisley, UK) and then in type II collagenase 1.4 g/mL in Dulbecco's Modified Eagle's Medium (DMEM, Sigma-Aldrich, St. Louis, MO, USA). Then bone samples were placed in 25 cm^2^ culture flasks containing DMEM supplemented with 20 % Foetal Bovine Serum (FBS, PAN-Biotech GmbH, Aidenbach, Germany) and 1 % Penicillin/Streptomycin (PS, Thermo Fisher Scientific, Waltham, MA, USA), incubated at 37°C and 5 % CO_2_ to allow cell migration. Cells were then expanded in DMEM supplemented with 10 % FBS and 1 % PS. At the third passage, cells were used either for bone recellularization or to collect the supernatants. For this study, primary human osteoblasts cultures were made from 3 donors (622, 623 and 635) and used both for recellularization and conditioned culture media processing.

### 2.4 Bone recellularization

Primary osteoblasts were seeded onto frozen bone discs at a density of 20 000 cells/mL and incubated for 3 days at 37°C and 5 % CO_2_. The culture medium was then refreshed, and after an additional day of incubation, recellularized bone discs were brought into contact with bacteria. Cell-free control (referred to as the “control”) were performed simultaneously and under the same culture condition. A scanning electron microscopy (SEM) control was performed to verify bone cellularization.

### 2.5 Supernatants

Primary osteoblasts were cultured to 60 % confluence (assessed visually by microscopy) then washed with PBS and incubated with DMEM supplemented with 10 % FBS without antibiotics for 72 h. The collected supernatants were then filtered using 0.22 μm filter and stored at −80°C. The control medium (DMEM supplemented with 10 % FBS without antibiotics), was also collected and filtered the same way.

### 2.6 *C. acnes* planktonic growth and adhesion

Bacterial counts were performed on both the planktonic part and the adherent part. To determine the quantity of adherent bacteria, bone explants and/or titanium discs were washed with PBS 1X and transferred to a 15 mL-tube containing 2 mL of PBS 1X, and bacteria were collected before and after 5 min in ultrasonic bath (40 kHz). The dilutions of interest were then seeded on blood agar plates using automatic seeder EasySpiral (Interscience, Saint-Nom-la-Bretèche, France) and incubated 5 days under anaerobic atmosphere. The number of recovered colony-forming units (CFU) was obtained using automatic counter SCAN 1200 (Interscience, Saint-Nom la-Bretèche, France) and the quantity of live adherent bacteria was determined as follows: [CFU/mL after ultrasonic bath] – [CFU/mL before ultrasonic bath]. Density of adherent bacteria (per cm^2^) on each explant was then calculated.

The percentage of adherent bacteria was calculated as [adherent bacteria ÷ total bacteria] × 100, with total bacteria being the sum of planktonic and adherent bacteria.

Bone explants from at least 10 donors were used for tests of *C. acne*s assays on bone explants, while 6 donors were dedicated to the tests of the impact on *C. acnes* adhesion on TF.

### 2.7 Histology staining and microscopic observations

After 5 days of incubation under anaerobic conditions with dBHI, fresh and frozen bone explants with or without bacteria were washed twice with PBS 1X, fixed in formaldehyde (VWR, Radnor, Pennsylvania, USA) 4 % and dehydrated by successive 1 h baths in graded ethanol solutions (70 %, 80 %, twice 95 % and thrice 100 %). Then, alcohol was replaced by xylene (two baths of 1 h) and samples were embedded in resin following the manufacturer's recommendations of Technovit® 9100 kit (Heraeus, Illange, France). Five microns serial sections were sectioned using HM355S microtome (Thermo Fisher Scientific, Waltham, MA, USA) and each bone sample was characterized after Masson trichrome staining (VWR, Radnor, Pennsylvania, USA) and observation using an Axiovert 200 M inverted microscope and the dedicated Axiovision v 3.2.6 software (Carl Zeiss, Oberkochen, Germany).

This experiment was performed with 2 different bone donor explants.

### 2.8 Scanning electron microscopy observations

Five days after incubation with *C*. *acnes* strains in dBHI under anaerobic condition, bone explants and TF discs were washed twice in PBS before to be fixed with 1 % (w/v) glutaraldehyde (Sigma-Aldrich, St. Louis, MO, USA) for 1 h, at room temperature. Then, surfaces were washed twice with sterile water for 10 min and dehydrated by successive 10 min baths in graded ethanol solutions (50 %, 70 %, 90 % and twice 100 %). TF discs were desiccated in a drop of hexamethyldisilazane (Sigma-Aldrich, St. Louis, MO, USA) and air-dried at room temperature. Bone explants were desiccated using the critical-point drying. Samples in 100 % ethanol were placed in acetone which was replaced by liquid carbon dioxide with several cycles under stirring, using the critical point dryer CPD 030 (BAL-TEC, Pfäffikon, Switzerland). Bones explants and TF discs were finally sputtered with a thin gold palladium film using the JEOL ion sputter JFC 1100 instrument. Bacteria were observed using the Scanning Electron Microscope JEOL JSM-7900F (JEOL, Tokyo, Japan). At least 2 different bone donor explants were used for this experiment.

### 2.9 Confocal scanning laser microscopy observations

After 5 days incubation, bone explants and TF discs were washed twice in PBS 1X and biofilms were stained using LIVE/DEAD BacLight Bacterial Viability kit (Molecular Probes, Eugene, OR, USA). SYTO-9™ (green fluorescence, all live and dead bacteria) was diluted at 100 μg/mL and propidium iodide (red fluorescence, damaged and dead bacteria) at 50 μg/mL in sterile NaCl 0.9 %. Fluorescents volumes (corresponding to the volume of biomass labeled with the fluorochrome and calculated using IMARIS software) of SYTO-9™ and PI were normalized to the total fluorescent volume to avoid the individual bone explant effect. *C. acnes* matrix biofilm on TF discs was labeled with concanavalin A (conA) Alexa Fluor™ 350-conjugated at 100 μg/mL (exopolysaccharides, Invitrogen, Waltham, MA, USA), TOTO-3™ iodide at 2 μM (eDNA, Invitrogen, Waltham, MA, USA) and SYPRO Ruby® (proteins, Invitrogen, Waltham, MA, USA). After 30 min of incubation at room temperature and in the dark, each support was washed twice in PBS 1X before to be transferred flipped upside-down in a Krystal 24-well plate with glass bottom (Porvair, UK). Biofilm was observed and imaged using confocal scanning laser microscopy (CSLM) LSM 710 NLO (Carl Zeiss, Oberkochen, Germany). The 3D-reconstruction and fluorescence volumes were obtained using IMARIS software. At least 4 bone donor explants were used.

### 2.10 mRNA expression of *C. acnes* genes

The expression of genes previously identified to be implicated in stress response and biofilm formation [22,23] was evaluated by mRNA analysis.

After 5 days incubation, planktonic and adherent bacteria, after ultrasonic bath, were collected, washed and pelleted independently by centrifugation (8 min, at 5000 g, 4°C) before being stored at - 80°C. Total RNAs were extracted using MasterPure™ RNA Purification Kit (Lucigen, Middleton, WI, USA) according to the manufacturer's protocol and 200 ng were then reverse transcribed into complementary DNA (cDNA) using High-Capacity cDNA Reverse Transcription Kit (Applied Biosystems, Waltham, MA, USA). Transcription products (10 ng) were amplified by qPCR using SYBR Takyon Mix (Eurogentec, Seraing, Belgium) on StepOne Plus™ system (Applied Biosystems) using primers detailed in Table 1. Results were analyzed with the StepOne™ software v2.3 (Applied Biosystems, Waltham, MA, USA) and the target transcript levels (N-target) were normalized to the housekeeping gene (*16SRNA*) transcript levels according to the following equation: N-target = 2^^ΔCt^, with ΔCt corresponding to the Ct value of the target gene after subtraction of Ct for the *16SRNA* gene.

**Table 1:** Nucleotide sequences of *C. acnes* genes primers used for qPCR.

	Gene	Function	Forward (5’→3′)	Reverse (5’→3′)
Housekeeping	*16SRNA*		TTATTGGGCGTAAAGGGCTC	CCCCTACCTTCCTCAAGTCA
Stress-related response	*lexA*	SOS response	CCAAGGAACGCGAAGTCTT	GCTGGACTGTTGATGTGCT
	*recA*		TCTATGGTCCCGAATCCTCC	TCACCAGCAGGGAATCTGTA
	*PPA0387*	Envelope stress response	AAGCTCCGCGAAACCATTAT	CAACCTGATCAGACTGGTCG
	*PPA0580*	Stress response	TCTACACCACCGAGGCTATT	CTTACCGCTACCACCCATCT
	*PPA1308*	Universal stress protein	CTCGACGCTTTCATGTCTGA	AAACATCCAGCGTTGATGGT
Biofilm ignition and formation	*PPA2127*	Adhesin	CACCTCAAGAAAGCAGCCA	CCAAGTGGGTGACGATACG
	*CAMP1*		TTCTACACCAAGACCAAGCTG	GCACGGCTCATGAAGTAAATG
	*dnaK*	Chaperonin	GGAGGTGTTCACTACTGCTG	GGCATAAGACCAGTCAGCTC
	*groEL*		CGTTACCAAGGACAACACCA	AGTCCGAGTTGTCGTACTCA
	*groES*		GACACCGCTAAGGAGAAGC	CGTCACCCTCCTTAACATCC
	*fusA*	Translation and proteins biosynthesis	TGAACTCGGTGCTCATTACG	CTCGAGGTAGAGCTCCATGA
	*smpB*		TATGCCTTCGGAACATGGC	GCCGTCCTTGAAGTAGAGC
	*tuf*		ATCTCGACAAGCCCTTCCT	TACCGACGATCTCGACCTC
	*fbaA*	Glycolysis	CAGGACCCGCTATTCAACTC	TCGATCTCGAGGATGAGCTT
	*eno*		CGAAGAAGCTTGGTGAGAAGA	AATCTGGTTCACCTTGACGAG
	*fumC*	TCA cycle	CCAAACCCCGACAAAATCAAG	TTCTTAGCGATCTTCGAAGCC
	*sucD*		ACCTACCAGCTCATGTACGA	GACGATGATGTCTGTCTCCG

This study was performed with at least 6 bone donor explants, identical to those used for bacterial enumeration, and 6 independent experiments for TF discs.

### 2.11 Graphical representation and statistical analysis

Results are represented as box-and-whisker plots with the box representing interquartile range, whiskers for the minimum and the maximum values, the bar inside the box for median value and point for each independent replicate. The average value is indicated by a red bar. The different variations between conditions were expressed from average values. The statistical significance was assessed using the exact 2-tailed non-parametric (owing to the lack of a normal distribution) Wilcoxon-Mann-Whitney test of GraphPad Prims (v8.0.1) software. Differences were considered significant at *p* < 0.05.

## 3 Results and discussion

The aim of this study was to determine whether the bone microenvironment modulates the response of *C. acnes* during colonization of osteoarticular prostheses. Two strains of different origins were investigated to assess whether they converge toward a common mechanism of adhesion and biofilm formation, or whether these processes are strain-dependent. Primary osteoblasts and bone explants from different donors were used to investigate the effect of bone and determine whether this effect was host-dependent.

### 3.1 The bone microenvironment modulates *C. acnes* adhesion to bone and biofilm initiation in a strain- and host-dependent manner

#### 3.1.1 *C. acnes* adheres to bone and initiates biofilm formation

*C. acnes* is known to adhere to titanium surfaces and to form biofilms [[8], [9], [10]]. The presence of *C. acnes* within OLCNs in a murine implant-associated osteomyelitis model [13] suggests that *C. acnes* is capable of colonizing bone. However, the ability of *C. acnes* to adhere directly to bone has not yet been thoroughly explored. Therefore, we used fresh bone explants to investigate *C. acnes* adhesion to bone (Fig. 1) and the regulation of gene expression in adherent bacteria compared with planktonic bacteria (Fig. 2).

**Fig. 1:**
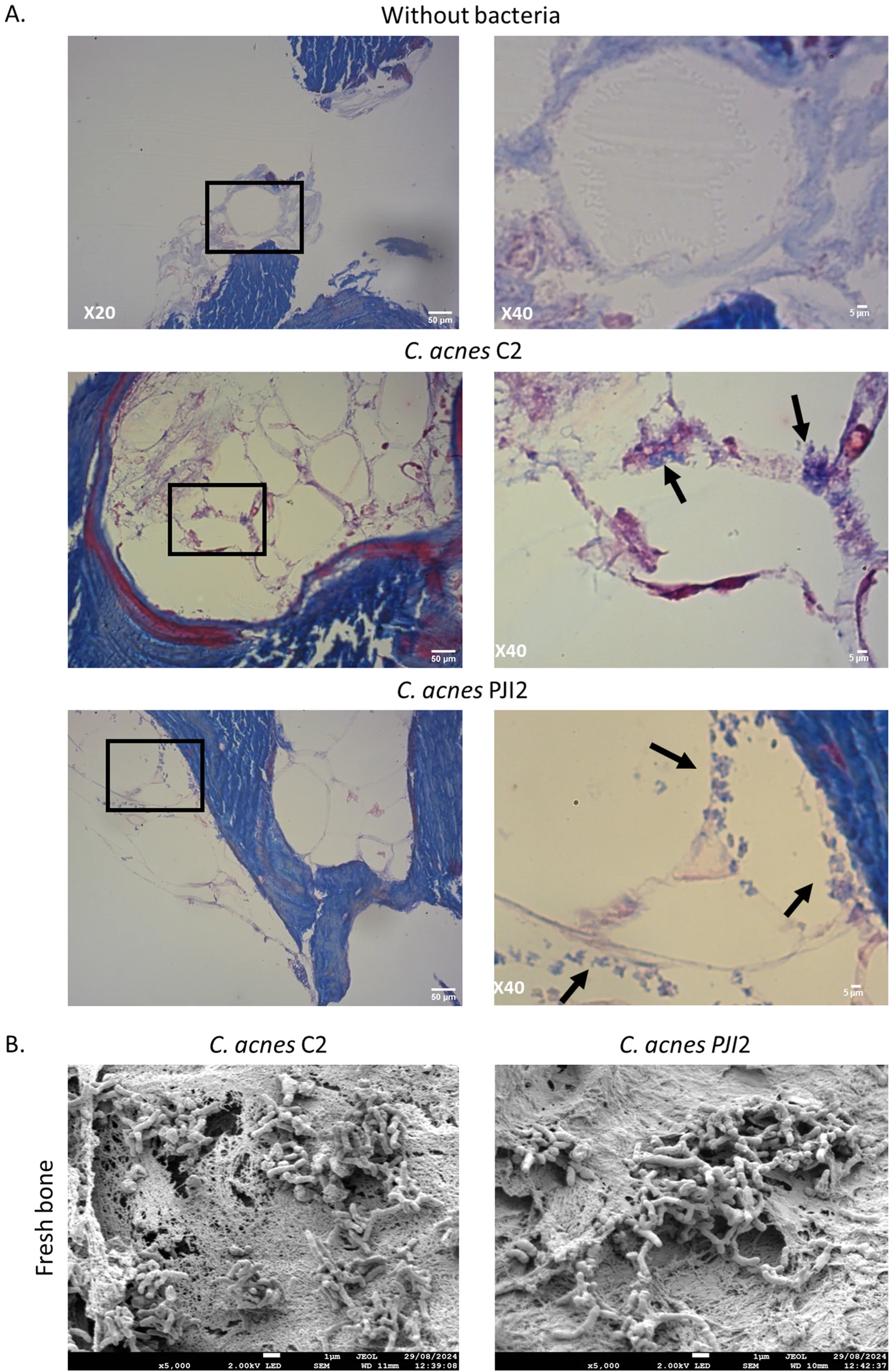
**Observation of bacterial adhesion on bone explant**. A. Observation of histological staining on top section of bone. Mature bone appears in blue, osteoid formation appears in red. Black arrows indicate bacterial clusters. Scale bar = 50 μm. Experiment was performed on explants from two donors. B. Morphology of bacteria and biofilm ignition on bone explant by SEM observation (magnification ×5,000). Scale bar = 1 μm. Experiment was performed on explants from three donors. (For interpretation of the references to colour in this figure legend, the reader is referred to the Web version of this article.)

**Fig. 2:**
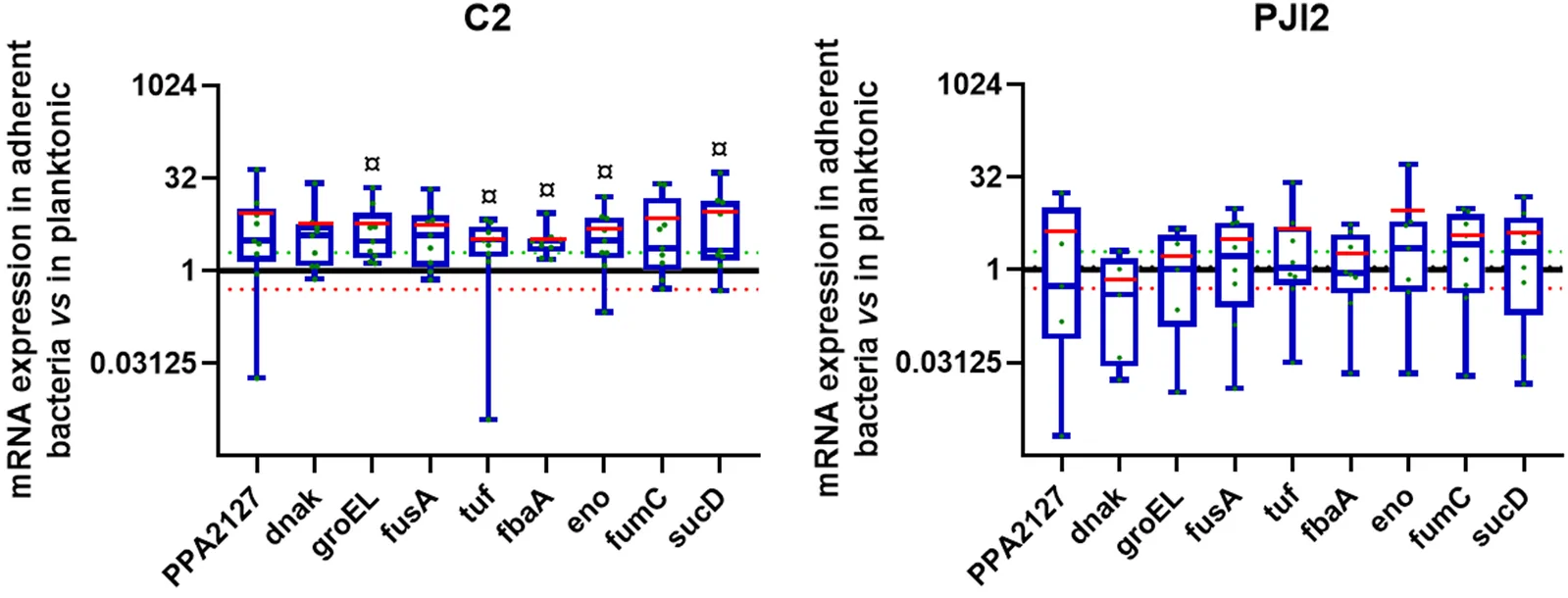
***C. acnes*' expression of genes involved in biofilm initiation and formation on fresh bone explant**. C2 and PJI2 genes expression when biofilm was formed on fresh bone. The red line represents the average value. The red dotted line represents the threshold for a 2-fold decrease in gene expression in the biofilm *versus* planktonic counterpart. The green dotted line corresponds to a 2-fold increase in gene expression. Experiment was performed on bone explants from at least 6 different donors. Wilcoxon-Mann-Whitney test: ¤ *p* < 0.05 for expression in adherent fraction compared with planktonic fraction. (For interpretation of the references to colour in this figure legend, the reader is referred to the Web version of this article.)

Histological analysis revealed no alteration in bone structure upon bacterial exposure with mineralized mature bone (in blue), osteoid undergoing formation or remodeling (in red), and the presence of bone lacunae (in light blue) (Fig. 1A). Specific structures (black arrows) were observed in the presence of both bacterial strains but not in the control condition, suggesting the formation of bacterial clusters (Fig. 1A). This result indicates that early colonization does not induce detectable tissue degradation under these conditions. For both *C. acnes* strains, bacterial adhesion to fresh bone was confirmed by SEM observations (Fig. 1B) and by quantification of adherent bacteria, with 6.5x10^6^ and 3.2x10^6^ adherent CFU/cm^2^ for C2 and PJI2 respectively (Supplementary Table 1).

These results confirm that *C. acnes* has the capacity to adhere to bone without degrading it.

As crystal violet staining of biofilm biomass and labeling of biofilm matrix components are not suitable methods due to absorption of the markers by bone, *C. acnes* biofilm formation was directly investigated by studying the expression of biofilm-related genes. In line with this biofilm-like formation on bone, five genes involved either in biofilm initiation (*groEL*, encoding a chaperonin) or in biofilm formation (*tuf,* protein biosynthesis; *fbaA* and *eno,* glycolysis; and *sucD*, TCA cycle) showed a significant increase in expression in adhered bacteria compared with their planktonic counterparts for the C2 strain (Fig. 2). However, a non-significant increase was observed for the other analyzed genes that are induced by environmental stress or involved in biofilm formation (Supplementary Fig. 1). For the PJI2 strain, a donor-dependent variability was observed, and no gene exhibited significantly altered expression (Fig. 2). Taken together, these results suggest that the adhesion of *C. acnes* to bone may trigger the development of a biofilm. A transcriptomic analysis using a bone model may therefore require identifying potential new genes activated during biofilm formation on bone.

#### 3.1.2 Bone cell presence strongly affects adhesion of the C2 strain

Then, we sought to unravel the role of bone components in *C. acnes* adhesion to bone. To address the role of bone cells, we first investigated the bacterial behavior on fresh bone compared with frozen bone, as freezing cycles of human tissues lead to cell death [Whittaker, 1984]; for example, the number of viable osteocytes within bone explants was drastically reduced (2.15 % of viable cells) after five 1-min liquid nitrogen freezing cycles [24].

The type of bone explant did not affect *C. acnes* planktonic growth (1.4x10^7^ and 1.8x10^7^ CFU/mL for C2 with frozen and fresh bone respectively, and 7.8x10^6^ and 1.2x10^7^ CFU/mL for PJI2) (Fig. 3A). However, a significant decrease in the number of adherent bacteria per cm^2^ was observed for C2 on frozen bone compared with fresh bone (3.3-fold decrease; Fig. 3B) and this decrease was also reflected in the percentage of adhesion (30.5 % on fresh bone versus 24 % on frozen bone; Fig. 3C). For the PJI2 strain, adhesion did not differ between fresh and frozen bone (Fig. 3B and C). These findings suggest that the presence of living bone cells might play a pro-biofilm role, at least on certain strains of *C. acnes*. CLSM observations revealed similar proportions of live and dead bacteria embedded in the biofilm for both strains in the presence of fresh and frozen bone explants (69.4 ± 34.7 % to 76.6 ± 12.7 % live bacteria) (Fig. 3D) suggesting that the biofilm formed on bone is mainly composed of living bacteria. Thus, bacterial adhesion to bone is strain-dependent, as C2 adhesion is affected by the presence of bone cells, whereas PJI2 adhesion is not.

**Fig. 3:**
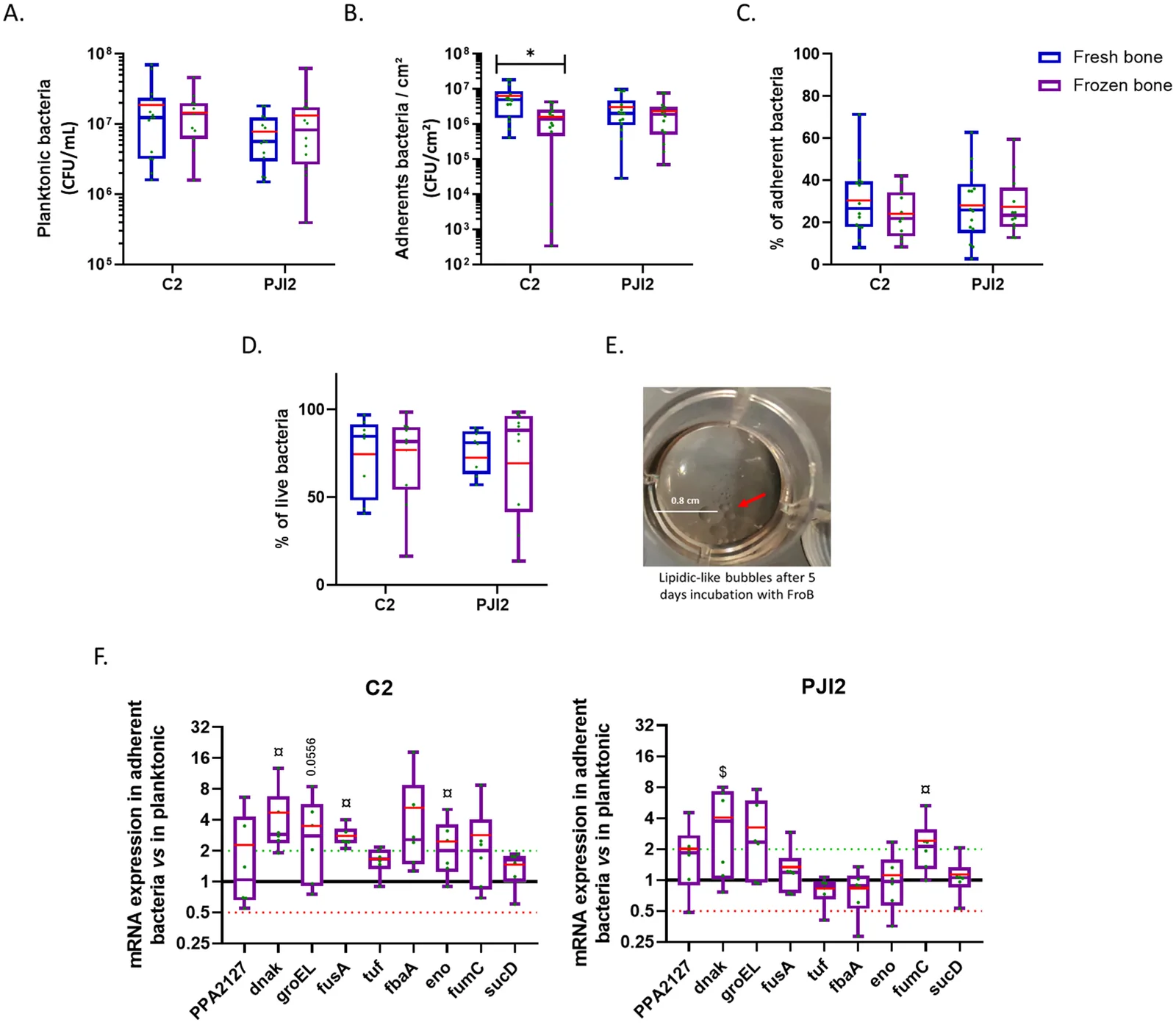
**Comparison of *C. acnes* behavior on fresh and on frozen bone explants**. A. Representation of the quantity of planktonic bacteria in presence of bone explants. B. Quantity of adherent bacteria embedded in biofilm per cm^2^, on bone explants. Percentage of adherent bacteria normalized by the total quantity of bacteria, on bone explant. Experiment was performed on bone explants from at least 10 different donors. D. Percentage of Live and Dead biofilm labelling on bone explant. Experiment was performed on bone explants from at least 4 different donors. E. Photography of lipidic-like bubbles released by bone explant (explant removed for the photography). F. C2 and PJI2 genes expression when biofilm was formed on frozen bone. The red dotted line represents the threshold for a 2-fold decrease in gene expression in the biofilm *versus* planktonic counterpart. The green dotted line corresponds to a 2-fold increase in gene expression. Experiment was performed on bone explants from 6 different donors. The red line represents the average value. Wilcoxon-Mann-Whitney test: *p < 0.05 between condition, ¤ *p* < 0.05 for expression in adherent fraction compared with planktonic fraction and $ p < 0.05 for gene expression on frozen bone compared with gene expression on fresh bone. (For interpretation of the references to colour in this figure legend, the reader is referred to the Web version of this article.)

In addition, we observed the presence of lipidic-like bubbles in the medium with frozen bone explants after 5 days of incubation (Fig. 3E). Considering that adipocytes are naturally present in bone, the presence of these bubbles could suggest adipocyte lysis. This process might release stored triglycerides, which could be hydrolyzed by lipases into free fatty acids (both saturated and unsaturated) and glycerol, providing substrates that could influence bacterial metabolism and biofilm formation [25]. Kim et al. demonstrated that fatty acids, including myristoleic acid, inhibited *Cutibacterium acnes* biofilm formation by reducing bacterial cell surface hydrophobicity [26]. Thus, the release of free fatty acids may also explain the lower bacterial adhesion to frozen bone observed here for the C2 strain.

Regarding *C. acnes* genes expression on frozen bone, a strain-dependent response was observed. Three genes (*dnaK*, *fusA* involved in proteins biosynthesis, and *eno,* involved in glycolysis) showed a significant increase in expression in adhered bacteria compared with their planktonic counterparts for the C2 strain (Fig. 3F). However, a non-significant increase in the expression of most of the other genes analyzed was also observed on frozen bone (Supplementary Fig. 1). For PJI2, only one gene, *fumC* (involved in the TCA cycle), was significantly upregulated in adherent bacteria compared with their planktonic counterparts, despite a similar trend for six other genes (Fig. 3F and Supplementary Fig. 1).

When comparing gene expression on frozen *versus* fresh bone, a decrease was observed for both strains (Supplementary Fig. 1). Interestingly, both strains showed a trend toward increased expression of *PPA0580*, which encodes a protein homologous to OsmC [Brüggeman et al., 2011]. OsmC is a peroxyreductase secreted in response to oxidative stress and bone cells are known to produce and release reactive oxygen species (ROS) [27]. This may explain the difference in PPA0580 expression between fresh and frozen bone.

Overall, while these findings suggest that *C*. *acnes* adhesion to bone is a strain-dependent response and bone explant status had a significant impact on the bacterial behavior, particularly at the level of the transcriptional response associated with biofilm initiation on bone. Indeed, the difference in cellularization and lipids presence between the two bone explant types may influence the bacterial adhesion. As osteoblasts can interact with *C. acnes* [18,28], it appeared important to investigate their direct involvement in bacterial adhesion to the bone.

#### 3.1.3 The osteoblasts play a role in adhesion of the C2 strain to bone

To confirm the impact of bone cells on *C. acnes* adhesion, we focused on investigating the influence of osteoblasts (Fig. 4). Primary osteoblasts were seeded on frozen bone explants for four days. SEM observations validated the presence of bone cells on the samples (Supplementary Fig. 2).

**Fig. 4:**
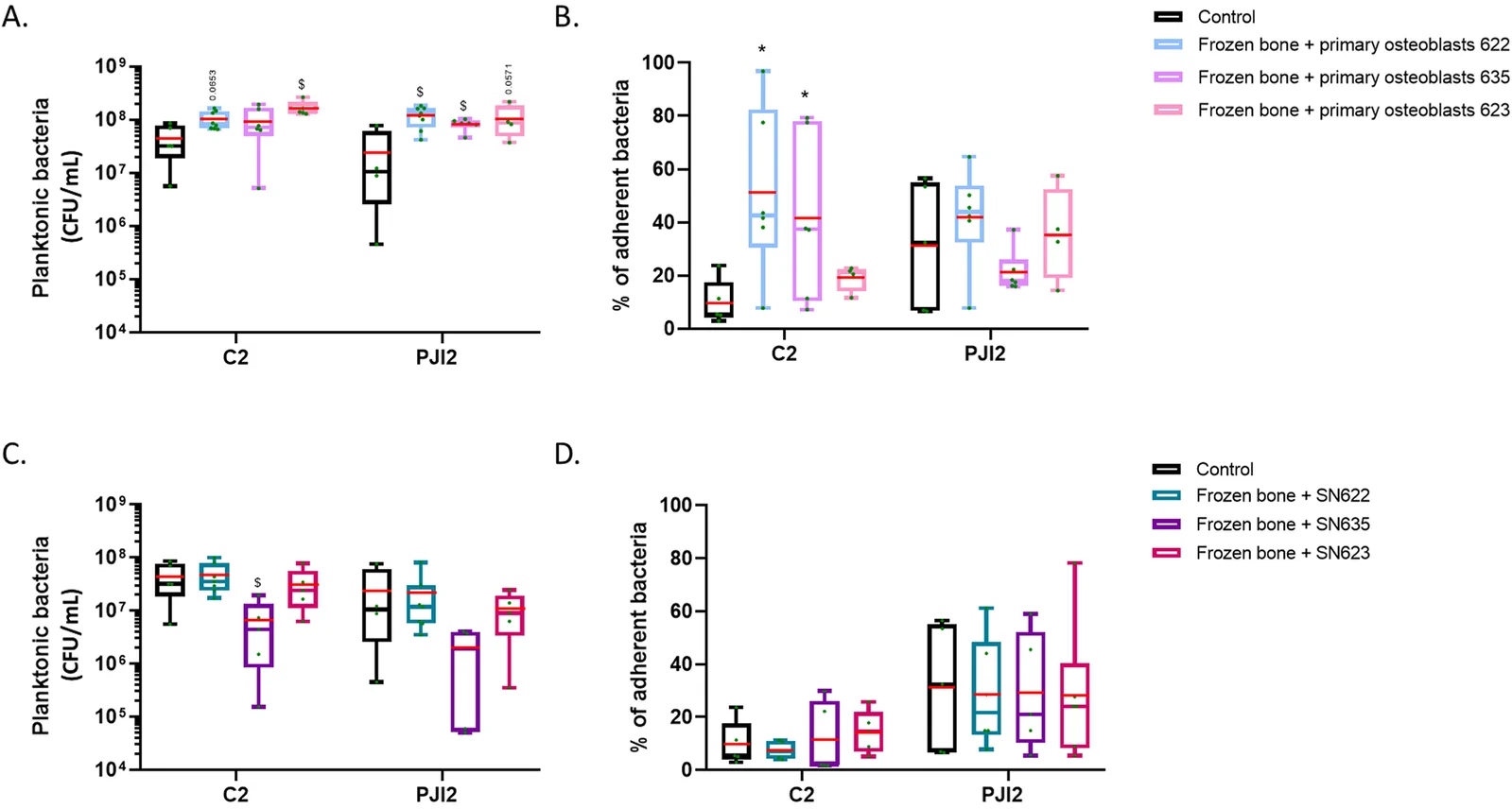
**Impact of bone cells and their secretions on bacterial adhesion and biofilm formation on frozen bone** A. Representation of the quantity of planktonic bacteria in presence of FroB with primary osteoblasts. B. Percentage of adherent bacteria normalized by the total quantity of bacteria on frozen bone with primary osteoblasts. C. Representation of the quantity of planktonic bacteria in presence of FroB with primary osteoblasts supernatants (SN). D. Percentage of adherent bacteria normalized by the total quantity of bacteria on frozen bone with primary osteoblasts supernatants (SN). The experiment was performed on bone explants from at least 5 different bone donors, using cells from three different bone donors (622, 635, 623). The red line represents the average value. Wilcoxon-Mann-Whitney $ *p* < 0.05 *versus* the control. (For interpretation of the references to colour in this figure legend, the reader is referred to the Web version of this article.)

The presence of osteoblasts on frozen bone increased both planktonic growth and adhesion for the two strains, with a strong donor-dependent effect (Fig. 4A). Beyond this inter-donor variability, a consistent pattern emerged: osteoblast-bacteria coculture appeared to drive C2 strains adhesion to bone, whereas cellular secretions did not. Specifically, the presence of osteoblasts from donor 622 or donor 635 induced a significant increase in the percentage of adhesion (5.2-fold and 4.3-fold, respectively) for the C2 strain (Fig. 4B), whereas cells from donor 635 tended to reduce adhesion of the PJI2 strain. Osteoblasts from donor 623 had no effect on either strain. These results indicate that osteoblasts can modulate bacterial behavior, with both strain-dependent and host-dependent effects. While the mechanism underlying *C. acnes* adhesion to bone is currently unknown, adhesion to osteoblasts is a phenomenon that has already been observed, notably through studies of *C. acnes* internalization by these cells [18,28].

To decipher what is pivotal between osteoblast presence and secretions, we investigated *C. acnes* interactions with osteoblast culture supernatants (SN) alone and no major effect on bacterial adhesion to bone was observed. However, a donor-dependent effect was observed on overall bacterial growth. A significant inhibition of C2 planktonic growth was observed upon exposure to primary osteoblast supernatant 635 (6.8-fold reduction), without affecting the percentage of adherent bacteria relative to the total quantity of bacteria (Fig. 4C and D). Similar effects were observed for the PJI2 strain, with a 12.4-fold reduction in planktonic bacteria (Fig. 4C). Such an effect might be explained the ability of bone cells to secrete antimicrobial peptides, such as β defensins [Varoga et al., 2008], which may impact *C. acne*s global growth. As osteoblasts were not in contact with bacteria prior to supernatant collection, these results suggest that bone cells, specifically osteoblasts, secrete patient-specific factors that may play a role in the development of PJIs.

Consequently, we hypothesized that the differences in adhesion observed between fresh bone and frozen bone are more likely driven by direct bacteria–cell interactions rather than by soluble factors.

Altogether, these results indicate that *C. acnes* adheres to bone and activates biofilm-related genes in a strain-dependent manner. Bone cells, and particularly osteoblasts, modulate bacterial adhesion in both a strain- and donor-dependent response, especially with cell-bacteria coculture. In contrast, cellular secretions alone have no effect on adhesion. Indeed, the presence of osteoblasts enhances adhesion of the C2 strain to bone, whereas the adhesion of the PJI2 strain appears to be less affected by the presence or absence of bone cells.

### 3.2 The bone microenvironment modulates *C. acnes* colonization of titanium in a strain-dependent manner

#### 3.2.1 Bone matrix and bone cells participate to *C. acnes* adhesion and biofilm formation on titanium

To investigate the effect of bone on *C. acnes* colonization of titanium, fibronectin-coated titanium discs (TF) were first co-incubated with either fresh or frozen bone explants, in the presence of *C. acnes.* In a second set of experiments, the effect of osteoblast-derived secretions was assessed by co-incubating TF with frozen bone explants and osteoblast-conditioned supernatants. Bacterial counts were performed on both bone and the implant. In this section, only the results obtained on TF are presented, as no significant difference of bacterial adhesion to bone (fresh or frozen) were observed between conditions with or without TF (Supplementary Table 1).

As expected, adherent *C. acnes* were observed on the titanium, both in the presence and absence of bone explants (Fig. 5). However, the presence of bone altered bacterial morphology, with a significant increase in bacterial length (yellow arrow) for the C2 strain. Bacterial elongation has frequently been associated with dysregulation of the cell division protein FtsZ [29], which has been identified in members of the phylum *Actinobacteria* [Kraxner et al., 2019] as well as in the genome of *Cutibacterium acnes* subsp. *defendens* (ATCC 11828) [30]. Cell division is tightly regulated by the bacterium's metabolic state and nutrient availability [Monahan et Harry, 2015; [31]. The elongation observed in the bacterial strain C2 may result from molecules produced by bone cells from the bone explant, which could interact directly or indirectly with effectors that regulate FtsZ. Similar phenomena have been reported during intracellular infection with *Mycobacterium tuberculosis*, where host-cell environments disrupt FtsZ ring assembly and lead to bacterial filamentation [32]. Interestingly, no effect on bacterial length was observed for the PJI2 strain despite the presence of the *ftsZ* gene in its genome (unpublished data), highlighting that the impact of the bone environment on the establishment of bacterial infection may vary depending on the strain (origin, genotype).

**Fig. 5:**
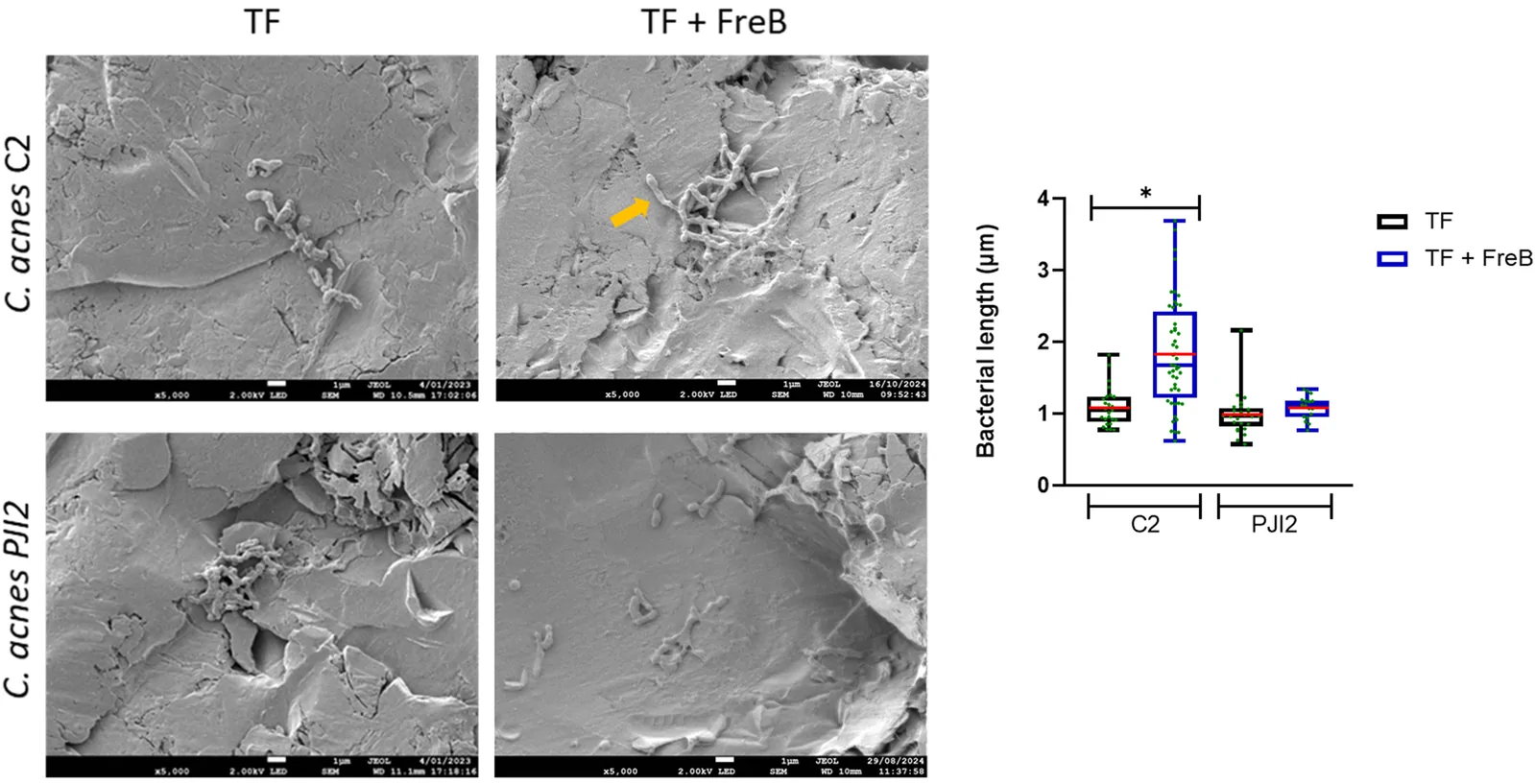
**Effect of fresh bone explant on *C. acnes* length on TF for the C2 strain** Morphology and length of bacteria on TF with or without bone explant assessed by SEM observation (magnification ×5,000). Scale bar = 1 μm. Yellow arrow shows elongated *C. acnes* on TF. The red line represents the average value Experiment was performed on bone explants from 2 different donors. (For interpretation of the references to colour in this figure legend, the reader is referred to the Web version of this article.)

The effects of fresh or frozen bone were examined, and the results were presented in a single figure to enable direct comparison between both types of explant (Fig. 6 and Supplementary Fig. 3).

**Fig. 6:**
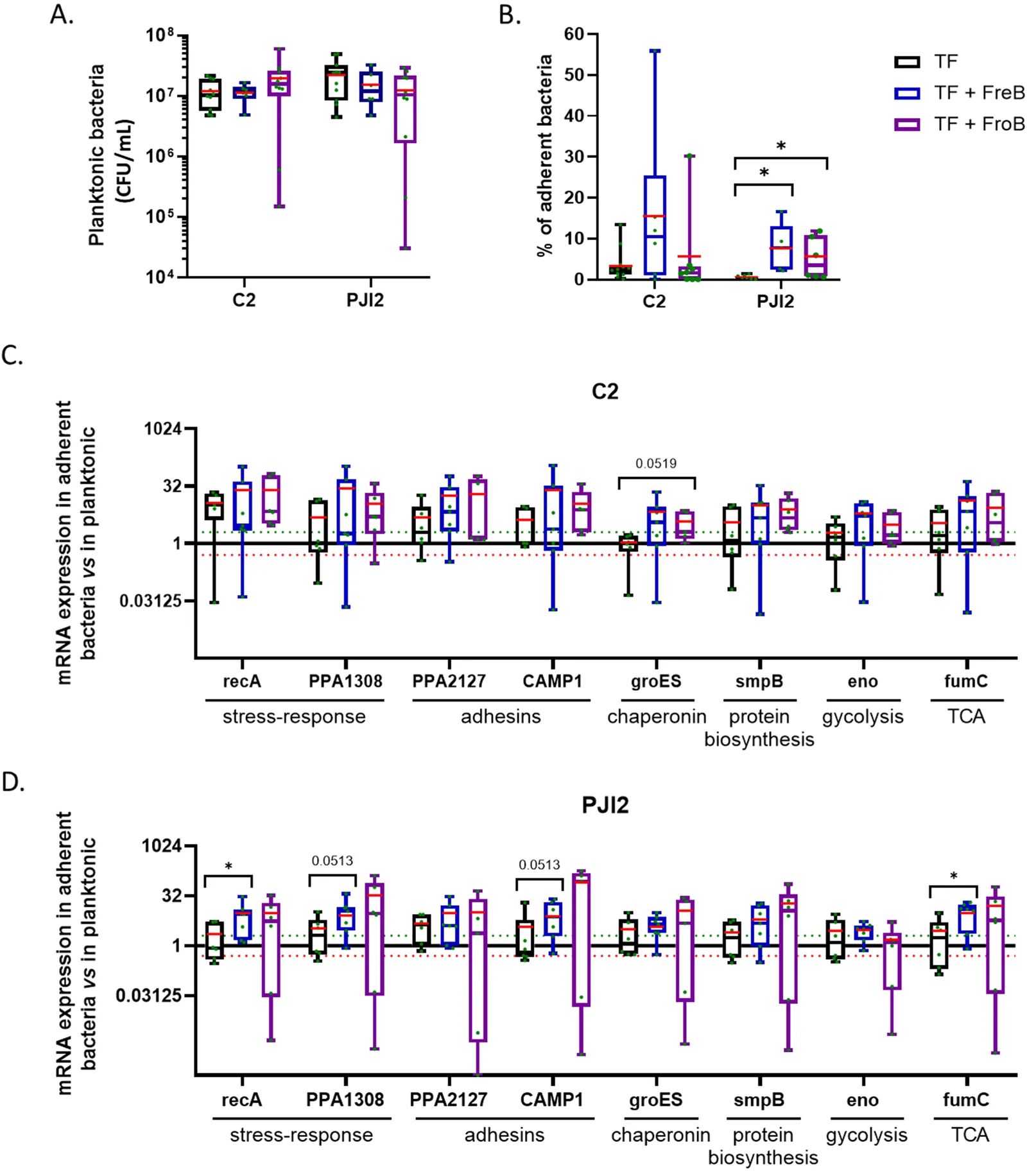
**Effect of bone explant on *C. acnes* adhesion and biofilm formation on TF** A. Quantity of planktonic bacteria in presence of TF. B. Percentage of adherent bacteria normalized by the total quantity of bacteria on TF discs in presence of fresh bone or frozen bone. Experiment was performed on bone explants from at least 6 different donors. C. Expression of genes when biofilm was formed on TF (black), on TF with fresh bone (blue) and on TF with frozen bone (purple) for C2 and D. for PJI2. Experiment was performed on bone explants from at least 6 different donors. The red line represents the average value. Wilcoxon-Mann-Whitney *p < 0.05 between condition. (For interpretation of the references to colour in this figure legend, the reader is referred to the Web version of this article.)

##### 3.2.1.1 Cellular bone increases *C. acnes* adhesion but induces strain-dependent activation of biofilm-associated genes

While the presence of fresh bone explant did not affect the quantity of planktonic bacteria (Fig. 6A), it increased *C. acnes* adhesion to the TF discs with adhesion percentage of 15.6 % (*versus* 3.6 % without FreB) for C2, and 7.7 % (*versus* 0.6 % without FreB) for PJI2 (Fig. 6B). Thus, the presence of fresh bone explants enhanced *C. acnes* adhesion on titanium.

Gene expression analysis revealed a strain-dependent response. For the C2 strain, most genes involved in stress response and biofilm formation showed a slight, non-significant increase in expression in the presence of FreB compared with bone-free conditions (Fig. 6C and Supplementary Fig. 3). For PJI2, several genes were upregulated in the presence of FreB, with *recA* (x4.5-fold increase) and *fumC* (x3.9-fold increase) showing significant increases (Fig. 6D and Supplementary Fig. 3). Thus, fresh bone appeared to induce different stress responses depending on the strain: oxidative stress in C2 and an SOS response in PJI2.

Whether in the presence or absence of fresh bone, the fluorescent signals of live and dead bacteria in biofilms formed on TF discs were similar for both strains (Supplementary Fig. 4). Analysis of the biofilm matrix showed no significant differences in fluorescence signals. For both strains, the biofilm remained primarily composed of proteins and polysaccharides, although a tendency towards an increase in eDNA was observed for the C2 strain in the presence of fresh bone (2.8-fold increase) (Supplementary Fig. 4). The observed increase in eDNA could result from enhanced bacterial cell death induced by stress mediated through the presence of bone cells.

Overall, the presence of fresh bone enhances *C. acnes* adhesion to titanium and partially induces a strain-dependent transcriptional response of biofilm-associated genes without significantly affecting the biofilm matrix composition. In the presence of fresh bone, C2 adhesion to titanium, as well as its morphology, is altered, whereas PJI2 does not exhibit any phenotypic changes, despite an overexpression of biofilm-associated genes.

##### 3.2.1.2 Acellular bone influences both adhesion and biofilm formation on titanium for PJI2

While previous experiments demonstrated that fresh bone impacted *C. acnes* adhesion to titanium, it remained unclear whether this effect was due to the bone cells, their secretions, or the bone matrix. To understand how bone affects the behavior of *C. acnes* on titanium, frozen bone explants were also used to test the effect of the bone matrix alone.

The presence of frozen bone did not affect the quantity of planktonic bacteria for the two strains (Fig. 6A). However, a significant increase of bacterial adhesion on titanium was observed for the PJI2 strain with an adhesion percentage of 5.1 % (*versus* 0.6 % without FroB) (Fig. 6B). No significant difference in gene expression was observed in the presence of frozen bone. Nevertheless, most genes tended to be upregulated in adherent bacteria compared with their planktonic counterparts in the presence of frozen bone for both C2 and PJI2 strains (Fig. 6C and D and Supplementary Fig. 3). Compared with gene expression on TF alone, a decrease in expression was observed for *fbaA* and *eno*, involved in glycolysis, as well as for *fusA* for the PJI2 strain.

One possible explanation for these observations is that the mineral phase of the bone matrix, composed predominantly of calcium and phosphate salts, undergoes dissolution during bone remodeling and resorption, resulting in the release of ions such as Ca^2+^ and PO_4_^3−^ into the extracellular environment [33]. Although no study has specifically addressed this topic, freeze–thaw cycles of bone explants could damage the bone mineral matrix, leading to prolonged ion release and osmotic stress. In response to osmotic or nutrient stress, the synthesis of the alarmones (p)ppGpp by RelA/SpoT and the induction of the general stress sigma factor RpoS in bacteria lead to widespread transcriptional reprogramming and down-regulation of growth-associated pathways such as glycolysis [34,35].

In the presence of frozen bone, a significant decrease in protein content was also observed on TF discs (x4.9-fold reduction) for the C2 strain, while no significant difference was detected for PJI2 (Supplementary Fig. 4). This result may also be associated with alterations in the bone matrix caused by freeze-thaw cycles, such as denaturation of matrix proteins, release of intracellular contents, but also with strain-specific properties.

To summarize, frozen bone, which ultimately represents the bone matrix, promotes PJI2 adhesion to titanium and tends to increase the expression of biofilm-associated genes, without affecting biofilm matrix composition. In contrast, frozen bone does not affect C2 adhesion but alters its matrix composition. These findings further support a strain-dependent response to the bone microenvironment and highlight the respective roles of the bone matrix and bone cells.

#### 3.2.2 Osteoblast secretions play a role in modulating bacterial adhesion to titanium

*C. acnes* strains were finally cultivated in the presence of frozen bone explants with the addition of primary osteoblasts supernatants, to assess the impact of cellular secretions on bacterial adhesion to titanium.

The presence of frozen bone and primary osteoblasts supernatants had no significant impact on *C. acnes* planktonic growth for both C2 and PJI2 (Fig. 7A), indicating that these conditions do not influence bacterial proliferation. However, their impact on adhesion to TF discs was both strain-dependent and donor-dependent: a significant increase of bacterial adhesion was observed for C2 in the presence of supernatant 622 (SN622) whereas a significant increase of bacterial adhesion was observed for PJI2 in the presence of the other two supernatants (SN635 and SN623) but not in the presence of SN622 (Fig. 7B). Previously, we showed that supernatants from primary osteoblast donors had no effect on *C. acnes* growth and adhesion to bone, except for SN635, which inhibited planktonic growth. However, osteoblast secretions increased *C. acnes* adhesion to TF. These results suggest that soluble compounds derived from osteoblasts may enhance bacterial adhesion to TF, possibly through indirect interactions with fibronectin or by modulating the extracellular matrix environment.

**Fig. 7:**
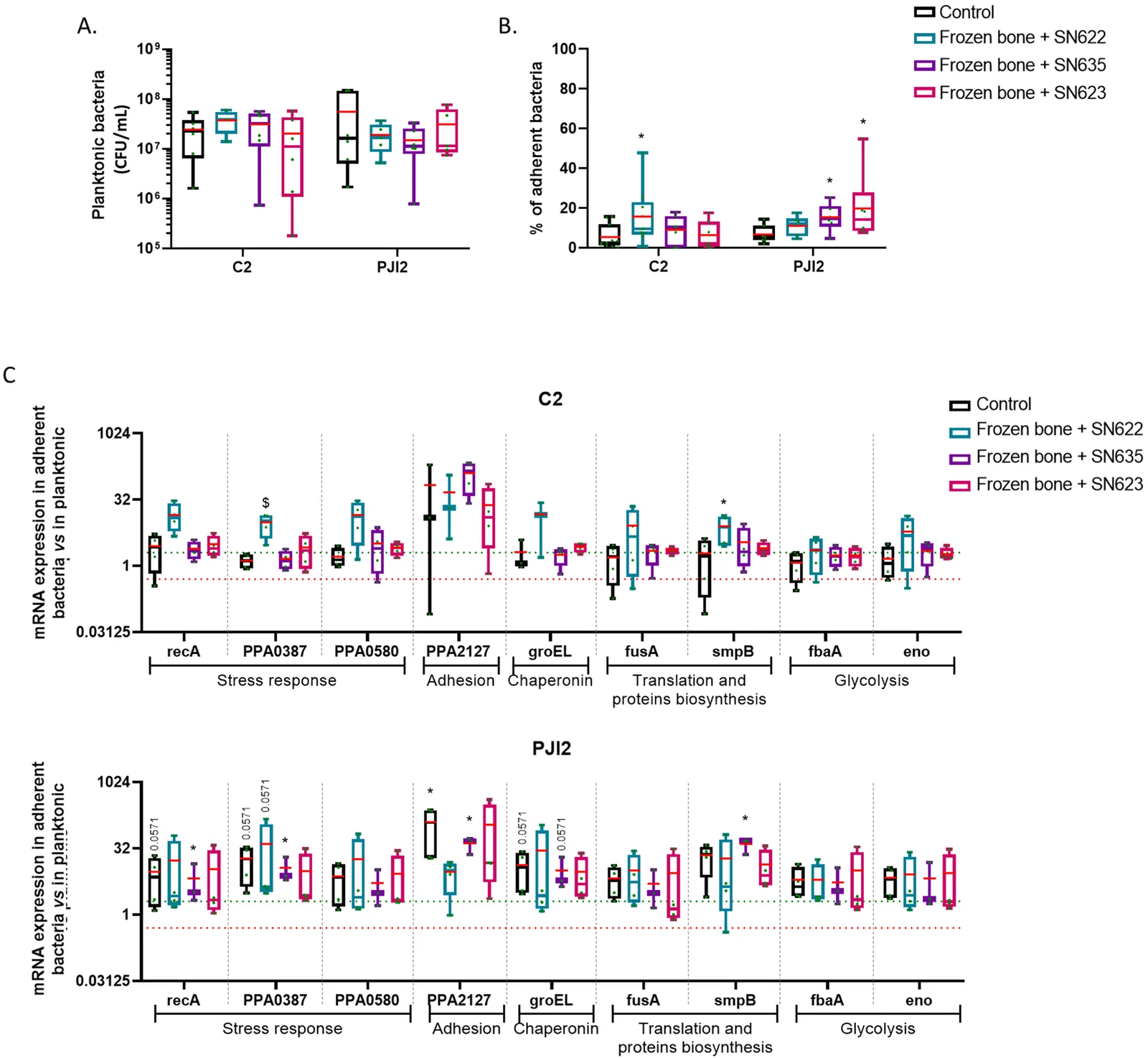
**Impact of primary osteoblast supernatants, with frozen bone explant, on bacterial growth and adhesion on TF disc** A. Quantity of planktonic bacteria in presence of on TF discs with frozen bone and primary osteoblasts supernatants. B. Percentage of adherent bacteria normalized by the total quantity of bacteria on TF discs with frozen bone and primary osteoblasts supernatants. The control is composed of 50 % osteoblast cell culture and 50 % dBHI. Experiment was performed on bone explants from at least 5 different donors. The red line represents the average value. Wilcoxon-Mann-Whitney *p < 0.05 *versus* control. C. mRNA expression of stress-induced genes and genes involved in biofilm initiation and formation of *C. acnes* on TF with frozen bone and primary osteoblasts supernatants. Experiment was performed at least 3 independent times. Wilcoxon-Mann-Whitney: biofilm *versus* planktonic, *p < 0.05 and SN *versus* control, $ *p* < 0.05. (For interpretation of the references to colour in this figure legend, the reader is referred to the Web version of this article.)

Gene expression in adherent bacteria was compared in the presence of supernatants *versus* control (cell-free medium) (Fig. 7C and Supplementary Fig. 5). For C2, interestingly, significant changes were observed only for *recA* and *PPA0387* in response to SN622, suggesting activation of an environmental stress response. In contrast, exposure to SN635 led to a marked upregulation of *PPA2127*, which encodes an adhesin, indicating potential enhanced adhesion. The SN623 triggered a broad increase in the expression of genes across multiple metabolic pathways, pointing to global metabolic adaptation. For PJI2, a significant decrease in *PPA2127* expression was observed in the presence of SN622. No significant changes were induced by SN635, although a general trend toward increased expression across multiple genes was noted. Exposure to SN623 resulted in decreased expression of numerous genes, reaching statistical significance for *PPA0387*, *groEL*, and *fumC*. These results correlate with those obtained for bacterial adhesion, with a particularly pronounced effect of SN622 on the C2 strain and of SN635 on the PJI2 strain, suggesting that strong biofilm induction depends on a complex strain-host relationship. They further highlight that different supernatants elicit strain-specific transcriptional responses, potentially reflecting distinct environmental cues and survival strategies.

Live/dead staining of biofilms formed on TF in the presence of frozen bone revealed a donor-dependent effect of the supernatants on the proportion of dead bacteria for the PJI2 strain, particularly with SN623, which showed a trend toward a 2-fold increase compared with the control (Supplementary Fig. 6). The increased cell death in the presence of SN623 may reflect autolysis releasing eDNA, a key component for biofilm initiation and formation [36], and may suggest that biofilm development is more advanced with this donor compared with the other donors.

Altogether, these results showed that bone cells secretions can influence *C. acnes* adhesion on TF disc. Osteoblasts secrete pro-inflammatory cytokines and chemokines (IL-6, IL-1β, TNF-α, IL-8, etc.) that may influence bacterial adhesion [37]. For example, the presence of chemokines CXCL9 and CXCL10 in clinical samples from *S*. *aureus* infections has been shown to induce the release of the virulence factor protein A (SpA), which is involved in *S. aureus* biofilm formation [38]. Osteoblasts also synthesize components of the bone extracellular matrix, including fibronectin, type I collagen, osteopontin, and bone sialoprotein [Maciel et al., 2023], which can potentially interact with bacterial surface adhesins. Finally, osteoblasts may secrete antimicrobial peptides, including β-defensins [Varoga et al., 2009]. At low concentrations, these antibacterial molecules may induce an adverse effect, as they can promote biofilm formation and thereby increase the risk of treatment recalcitrance. As previously observed, the antimicrobial effect of some supernatants can increase the quantity of dead bacteria leading to a release of eDNA, which is necessary to the biofilm initiation and formation [36].

In summary, our results indicate that the bacterial response to bone explants varies depending on both the bacterial strain and the host. Strain-dependent variation may reflect inherent genetic differences between strains, differential sensing of the bone environment, or the activation of strain-specific survival strategies. Notably, the C2 strain exhibited stronger adhesion in the presence of fresh bone and initiated biofilm formation primarily in response to oxidative stress, whereas the PJI2 strain was affected by the presence of bone (with or without cells) and initiated biofilm formation in response to DNA damage, consistent with an SOS-like response. This indicates that biofilm initiation could arise through different stresses related to bone microenvironment, depending on strain sensitivity. Despite these differences in initiation cues, the overall composition of the resulting biofilm on titanium remained largely unchanged. These findings suggest that while the triggers for biofilm formation differ between strains, the final biofilm phenotype on an abiotic surface such as titanium may be more conserved. Lamret et al. also demonstrated a strain-dependent response to the presence of frozen bone on *S. aureus* adhesion to fibronectin-coated titanium, with SH1000 exhibiting reduced adhesion, downregulation of *sigB* expression, and altered biofilm composition, whereas no significant changes were observed for the USA300 strain [20]. Furthermore, the differences in the bacterial response of the two strains toward donors were clear, with one donor inducing biofilm formation in C2 (622) and the other two (623, 635) inducing it in PJI2. This observation highlights the complexity of this two-actor system influencing biofilm initiation in *C. acnes*-related PJIs and, consequently, the course of the infection.

## 4 Conclusion

Our findings identify the bone microenvironment as an active determinant of *C. acnes* adhesion and biofilm formation to the bone-implant interface. The presence of bone cells, rather than their secretions, plays a role in the adhesion of strain C2 to bone, while this effect is not observed for strain PJI2. This difference may reflect the naïve nature of the C2 strain to the bone environment, in contrast to the PJI2 strain. On the other hand, bone cell secretions appear to influence the adhesion of both C2 and PJI2 to titanium, which mimics the prosthesis. Similarly, components of the bone matrix also seem to enhance PJI2 adhesion to titanium, although this increase is less pronounced than that observed in the presence of bone cells.

Altogether, the present results show that *C. acnes* biofilm establishment emerges from interactions between bacterial strain, host bone microenvironment, and colonized implant surface, and suggest that the bone microenvironment, and especially bone cells, plays a key role in the establishment of *C. acnes* PJIs, but the effects can vary greatly depending on the host characteristics. Patient-derived cells therefore influence the behavior of individual strains, highlighting the difficulty (and even the lack of relevance) of predicting the course of the infection based solely on bacterial strain analysis. Moreover, the two strains displayed distinct responses to the bone environment, consistent with different stress-adaptation pathways, despite converging toward a broadly similar biofilm phenotype on titanium. The observed strain-dependent variability likely reflects genetic differences, distinct environmental sensing, and metabolic heterogeneity, leading to strain-specific survival strategies. To further explore these mechanisms, studies should include additional clinical *C. acnes* strains to allow grouping by similar susceptibilities and identification of bacterial biomarkers. Investigating osteoblast secretome from multiple donors may also reveal host-derived biomarkers and potentially suggest novel therapeutic avenues.

## CRediT authorship contribution statement

**Jennifer Varin-Simon:** Writing – review & editing, Writing – original draft, Visualization, Validation, Methodology, Investigation, Formal analysis, Data curation. **Christine Guillaume:** Methodology, Investigation. **Yasser Dghoughi:** Writing – review & editing, Methodology, Investigation. **Renaud Siboni:** Writing – review & editing, Methodology. **Frédéric Velard:** Writing – review & editing, Methodology, Investigation. **Sophie C. Gangloff:** Writing – review & editing, Validation, Supervision, Project administration, Funding acquisition. **Marius Colin:** Writing – review & editing, Validation, Supervision, Formal analysis, Data curation, Conceptualization. **Fany Reffuveille:** Writing – review & editing, Validation, Supervision, Resources, Project administration, Methodology, Funding acquisition, Formal analysis, Data curation, Conceptualization.

## Ethics declaration

Written informed consent to take part in the study and to publish the article has been obtained from all participants or their legal representatives. The privacy rights of participants have been observed.

This study included organ or tissue donors. This study includes human biological material and consent was obtained by donors, or their next of kin or legal representatives, for use in this study and for publication of the article. The samples used in this research were not sourced from executed prisoners or prisoners of conscience.

This study was performed in compliance with relevant laws, regulatory frameworks and guidelines where the research took place. This study was approved by the French Ministry of Research. (Approval No. DC-2014-2262)

## Declaration of competing interest

The authors declare no competing financial interests.

## Data Availability

Data will be made available on request.

## Abbreviations

BHI: Brain Heart Infusion
C. acnes: Cutibacterium acnes
ConA: Concanavalin A
CFU: Colony Forming Unit
CLSM: Confocal Scanning Laser Microscopy
DMEM: Dulbecco's Modified Eagle Medium
FBS: Fetal Bovine Serum
FreB: Fresh Bone
FroB: Frozen Bone
OLCN: Osteocytes Lacunar-Canalicular Networks
PBS: Phosphate Buffered Saline
PCR: Polymerase Chain Reaction
PI: Propidium Iodide
PJI: Prosthetic Joint Infection
*S. aureus*: *Staphylococcus aureus*
SEM: Scanning Electron Microscopy
TCA: Tricarboxylic Acid Cycle
TF: Titanium coated with Fibronectin

## References

[1] Shoji M.M., Chen A.F. (2020). Biofilms in periprosthetic joint infections: diagnostic modalities, current treatments, and future directions. J Knee Surg.

[2] Triffault-Fillit C., Ferry T., Laurent F., Pradat P., Dupieux C., Conrad A. (2019). Microbiologic epidemiology depending on time to occurrence of prosthetic joint infection. Clin Microbiol Infect.

[3] Patel A., Calfee R.P., Plante M., Fischer S.A., Green A. (2009). *Propionibacterium acnes* colonization of the human shoulder. J Shoulder Elb Surg.

[4] Tande A.J., Patel R. (2014). Prosthetic joint infection. Clin Microbiol Rev.

[5] Stewart P.S., Franklin M.J. (2008). Physiological heterogeneity in biofilms. Nat Rev Microbiol.

[6] Hall-Stoodley L., Costerton J.W., Stoodley P. (2004). Bacterial biofilms: from the natural environment to infectious diseases. Nat Rev Microbiol.

[7] Stewart P.S. (2015). Antimicrobial tolerance in biofilms. Microbiol Spectr.

[8] Bidossi A., Bottagisio M., De Grandi R., De Vecchi E. (2020). Ability of adhesion and biofilm formation of pathogens of periprosthetic joint infections on titanium-niobium nitride (TiNbN) ceramic coatings. J Orthop Surg Res.

[9] Garcia D., Mayfield C.K., Leong J., Deckey D.G., Zega A., Glasser J. (2020). Early adherence and biofilm formation of *Cutibacterium acnes* on spinal implant materials. Spine J.

[10] Varin-Simon J., Colin M., Velard F., Tang-Fichaux M., Ohl X., Mongaret C. (2024). *Cutibacterium acnes* biofilm formation is influenced by bone microenvironment, implant surfaces and bacterial internalization. BMC Microbiol.

[11] Yu J.L., Månsson R., Flock J.I., Ljungh Å. (1997). Fibronectin binding by *Propionibacterium acnes*. FEMS Immunol Med Microbiol.

[12] Arciola C.R., Campoccia D., Montanaro L. (2018). Implant infections: adhesion, biofilm formation and immune evasion. Nat Rev Microbiol.

[13] Botros M., de Mesy Bentley K.L., Schloemann D.T., Saito M., Constantine R., Ricciardi B.F., Muthukrishnan G. (2024). Cutibacterium acnes invades submicron osteocyte lacuno-canalicular networks following implant-associated osteomyelitis. J Orthop Res.

[14] Mainard D. (2011).

[15] Josse J., Velard F., Gangloff S.C. (2015). *Staphylococcus aureus* vs. osteoblast: relationship and consequences in osteomyelitis. Front Cell Infect Microbiol.

[16] Lemaire A., Varin-Simon J., Lamret F., Dubus M., Kerdjoudj H., Velard F., Gangloff S.C., Reffuveille F. (2023). *Staphylococcus aureus* behavior on artificial surfaces mimicking bone environment. Pathogens.

[17] Josse J., Laurent F., Diot A. (2017). Staphylococcal adhesion and host cell invasion: fibronectin-binding and other mechanisms. Front Microbiol.

[18] Aubin G.G., Baud’huin M., Lavigne J.P., Brion R., Gouin F., Lepelletier D. (2017). Interaction of *Cutibacterium (formerly Propionibacterium) acnes* with bone cells: a step toward understanding bone and joint infection development. Sci Rep.

[19] Dubus M., Varin J., Papa S., Rammal H., Chevrier J., Maisonneuve E. (2020). Interaction of *Cutibacterium acnes* with human bone marrow-derived mesenchymal stem cells. Acta Biomater.

[20] Lamret F., Lemaire A., Lagoutte M., Varin-Simon J., Abraham L., Colin M. (2023). Approaching prosthesis infection environment: development of an innovative in vitro *Staphylococcus aureus* biofilm model. Biofilm.

[21] Delion M., Braux J., Jourdain M.L., Guillaume C., Bour C., Gangloff S. (2016). Overexpression of RANKL in osteoblasts: a possible mechanism of susceptibility to bone disease in cystic fibrosis. J Pathol.

[22] Jahns A.C., Eilers H., Alexeyev O.A. (2016). Transcriptomic analysis of *Propionibacterium acnes* biofilms in vitro. Anaerobe.

[23] Achermann Y., Tran B., Kang M., Harro J.M., Shirtliff M.E. (2015). Immunoproteomic identification of in vivo–produced *Propionibacterium acnes* proteins in a rabbit biofilm infection model. Clin Vaccine Immunol.

[24] Jähn K., Braunstein V., Furlong P.I., Simpson A.E., Richards R.G., Stoddart M.J. (2010 May 10). A rapid method for the generation of uniform acellular bone explants: a technical note. J Orthop Surg Res.

[25] Zimmermann R., Strauss J.G., Haemmerle G., Schoiswohl G., Birner-Gruenberger R., Riederer M. (2011). Adipocyte lipolysis: from molecular mechanisms of regulation to disease and therapeutics. Nat Rev Mol Cell Biol.

[26] Kim Y.G., Lee J.H., Lee J. (2021). Antibiofilm activities of fatty acids including myristoleic acid against *Cutibacterium acnes* via reduced cell hydrophobicity. Phytomedicine.

[27] Marques-Carvalho A., Kim H.N., Almeida M. (2023). The role of reactive oxygen species in bone cell physiology and pathophysiology. BoneKEy Rep.

[28] Mongaret C., Varin-Simon J., Lamret F., El-Mahdy T.S., Brasme L., Vernet-Garnier V., Gangloff S.C., Ohl X., Reffuveille F. (2020). *Cutibacterium acnes* biofilm study during bone cells interaction. Microorganisms.

[29] Whitley K.D., Jukes C., Tregidgo N., Karinou E., Almada P., Cesbron Y., Henriques R., Dekker C., Holden S. (2021). FtsZ treadmilling is essential for Z-ring condensation and septal constriction initiation in *Bacillus subtilis* cell division. Nat Commun.

[30] Horváth B., Hunyadkürti J., Vörös A., Fekete C., Urbán E., Kemény L., Nagy I. (2012). Genome sequence of *Propionibacterium acnes* type II strain ATCC 11828. J Bacteriol.

[31] Galinier A., Foulquier E., Pompeo F. (2021). Metabolic control of cell elongation and cell division in *Bacillus subtilis*. Front Microbiol.

[32] Chauhan A., Madiraju M.V.V.S., Fol M., Lofton H., Maloney E., Reynolds R. (2006). Mycobacterium tuberculosis cells growing in macrophages are filamentous and deficient in FtsZ rings. J Bacteriol.

[33] Sugimoto K., Zhou Y., Peñaflor Galindo T.G., Kimura R., Tagaya M. (2023). Investigation of surface layers on biological and synthetic hydroxyapatites based on bone mineralization process. Biomimetics.

[34] Steinchen W., Bange G. (2020). ppGpp: magic modulators of bacterial physiology and metabolism. Nat Rev Microbiol.

[35] Zhang H., Yin Y., Li X., Zhou L., Wang J., Zhang Z. (2016). The roles of RelA/(p)ppGpp in glucose-starvation induced adaptive response in *Streptococcus suis*. Sci Rep.

[36] Kuehnast T., Cakar F., Weinhäupl T., Pilz A., Selak S., Schmidt M.A. (2018). Comparative analyses of biofilm formation among different *Cutibacterium acnes* isolates. Int J Med Microbiol.

[37] Saint-Pastou Terrier C., Gasque P. (2017 Oct). Bone responses in health and infectious diseases: a focus on osteoblasts. J Infect.

[38] Granata V., Possetti V., Parente R., Bottazzi B., Inforzato A., Sobacchi C. (2022). The osteoblast secretome in *Staphylococcus aureus* osteomyelitis. Front Immunol.

