# Supplementary material for: Bone cells modulate Cutibacterium acnes colonization and biofilm formation on bone and titanium
Source: Biofilm. 2026 Oct 5;12:100404. doi: 10.1016/j.bioflm.2026.100404 (PMC13672832; doi:10.1016/j.bioflm.2026.100404)
Supplement: Supplementary file 1 [file mmc1.docx]

**Supplementary Table 1: Average quantity of adherent bacteria on bone explants with or without TF**

|  |  | **FreB** | **FreB (+TF)** | **FroB** | **FroB (+TF)** |
| --- | --- | --- | --- | --- | --- |
| Adherent bacteria (CFU/cm²) | C2 | 6.52E+06 | 3.93E+06 | 1.98E+06 | 3.35E+06 |
|  | PJI2 | 3.19E+06 | 3.25E+06 | 2.46E+06 | 7.59E+06 |
