# Supplementary material for: Bone cells modulate Cutibacterium acnes colonization and biofilm formation on bone and titanium
Source: Biofilm. 2026 Oct 5;12:100404. doi: 10.1016/j.bioflm.2026.100404 (PMC13672832; doi:10.1016/j.bioflm.2026.100404)
Supplement: Supplementary file 2 [file mmc2.docx]

**Supplementary Figure 1: Heat-map of gene expression in adherent bacteria to bone explants relative to planktonic bacteria, based on average values across all donors.**

**Supplementary Figure 2: Observation of cellularization of frozen bone by SEM** (magnification x500 and x1,500). Scale bar = 10 µm. Red arrows show some osteoblasts seeded on frozen bone explant.

**Supplementary Figure 3: Heat-map of gene expression in adherent bacteria to TF without or with bone explants relative to planktonic bacteria, based on average values across all donors.**

**Supplementary Figure 4: *C. acnes* biofilm matrix composition on titanium coated with fibronectin.**

Fluorescent volumes of the matrix components: SYTO9™ for bacteria (green), Propidium Iodide (red) for dead bacteria, concanavalin A (conA, blue) for polysaccharides, TOTO-3 Iodide™ for extracellular DNA (eDNA, orange) and SYPRO Ruby® for proteins (dark red) were obtained using IMARIS software after acquisitions by CLSM. The red line represents the average value. Experiment was performed on bone explants from at least 3 different donors.

**Supplementary Figure 5: Heat-map of gene expression in adherent bacteria to TF with bone explants and osteoblasts supernatants relative to planktonic bacteria, based on average values across all donors.**

A high variability in response was observed for the PJI2 strain in the presence of SN635.

**Supplementary Figure 6: Percentage of dead/damaged bacteria in the biofilm on titanium coated with fibronectin after LIVE/DEAD staining.**

Percentage of Propidium Iodide in biofilm on TF alone or in presence of FroB, with or without primary osteoblast supernatant. The red line represents the average value. Experiment was performed on bone explants from at least 4 different donors
